# Supplementary material for: The Influence of Locality on Phenolic Profile and Antioxidant Capacity of Bud Extracts
Source: Foods. 2021 Jul 12;10(7):1608. doi: 10.3390/foods10071608 (PMC8306517; doi:10.3390/foods10071608)

**Table S1.** The main factories producing pollution in the surrounding of the monitored localities.

| | NOx | CO | $SO_2$ | CFCs | PM | HM | VOC |
|---|---|---|---|---|---|---|---|
| **Jičín** | | | | | | | |
| Prison Valdice | 2.036 | 0.065 | | | | | |
| Continental Automotive Czech Republic s.r.o. | 0.458 | 0.092 | | | | | 0.136 |
| Hydra a.s. | | | | | 0.156 | | 5.149 |
| KOBIT spol. s.r.o. | | | | | 0.019 | | 1.756 |
| | | | | | | | |
| **Hradec Králové** | | | | | | | |
| University Hospital | 4.254 | 0.428 | 0.159 | 0.079 | 0.018 | 0.0028 | |
| Mobile diesel generator | 0.082 | 0.018 | | | | | |
| Wastewater treatment plant | 1.693 | 2.525 | | | 0.070 | | |
| | | | | | | | |
| **Opatovice nad Labem** | | | | | | | |
| Thermal power station | 1703.5 | 322.7 | 1609.7 | 6.91 | 54.97 | 0.1294 | 0.00012 |

CFCs, Chlorofluorocarbons; PM, Particulate matter; HM, Heavy metals; VOC, Volatile organic compounds

**Jičín; GPS: 50°27'34"N 15°26'42"E**

The main sources of pollution: Prison Valdice (fuel burning), Continental Automotive Czech Republic s.r.o. (the production of electrical and electronic products for the automotive industry), Hydra a.s. (surface treatment of metal), KOBIT spol. s.r.o. (surface treatment of metal and plastic)

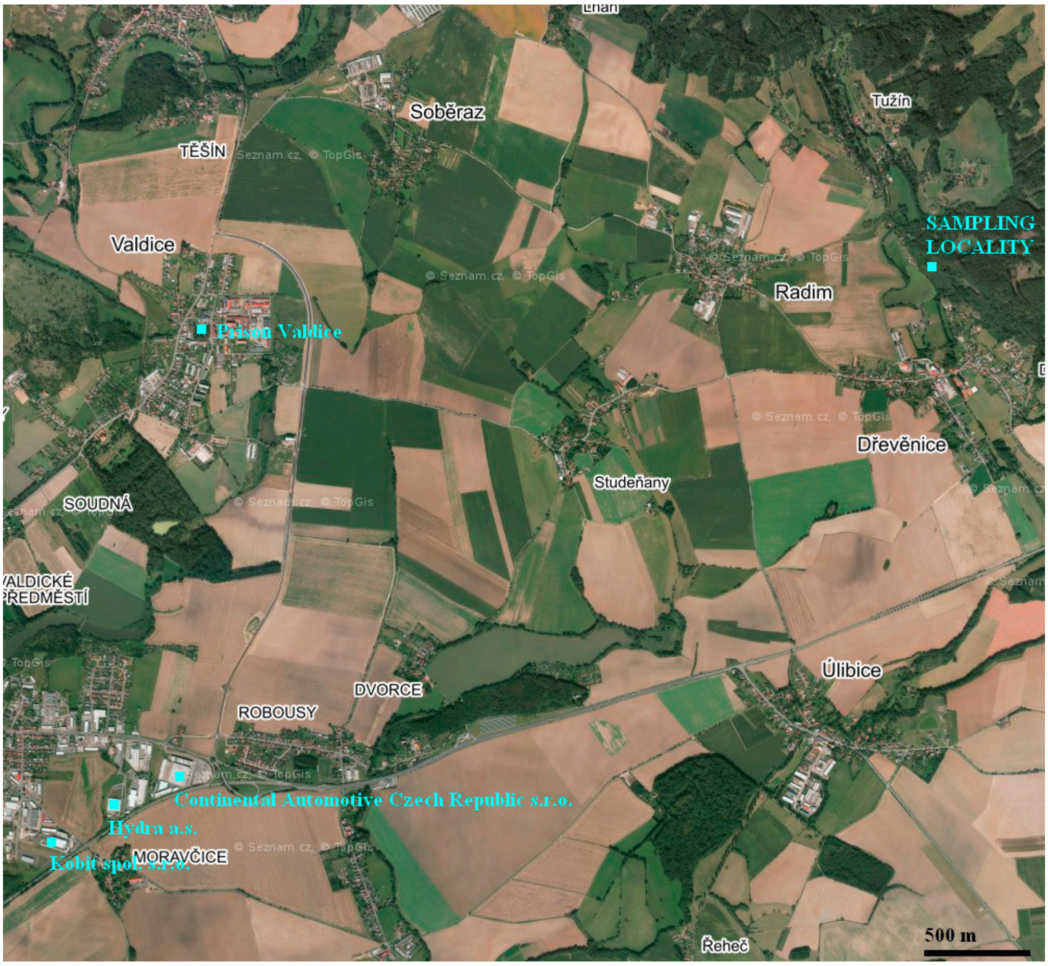

**Opatovice nad Labem; GPS: 50°7'10"N 15°47'30"E**
The main sources of pollution in this area is Thermal power station Opatovice, Inc.

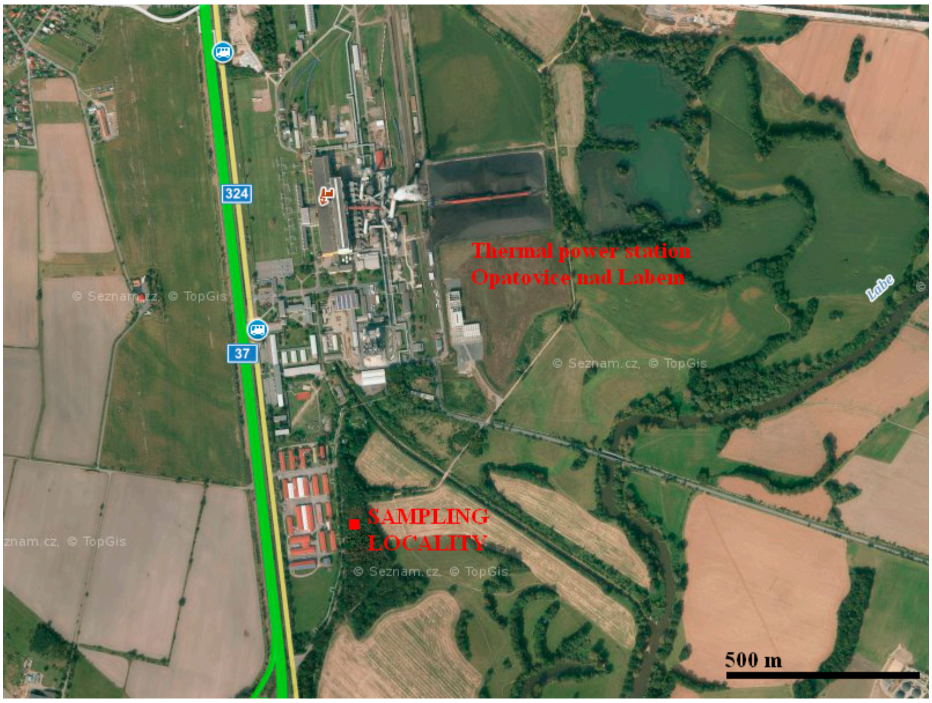


**Hradec Králové; GPS: 50°11'17"N 15°49'13"E**
The main sources of pollution: Universtiy Hospital, mobiel deisel generator, Wastewater treatment plant Hradec Králové, background traffic pollution

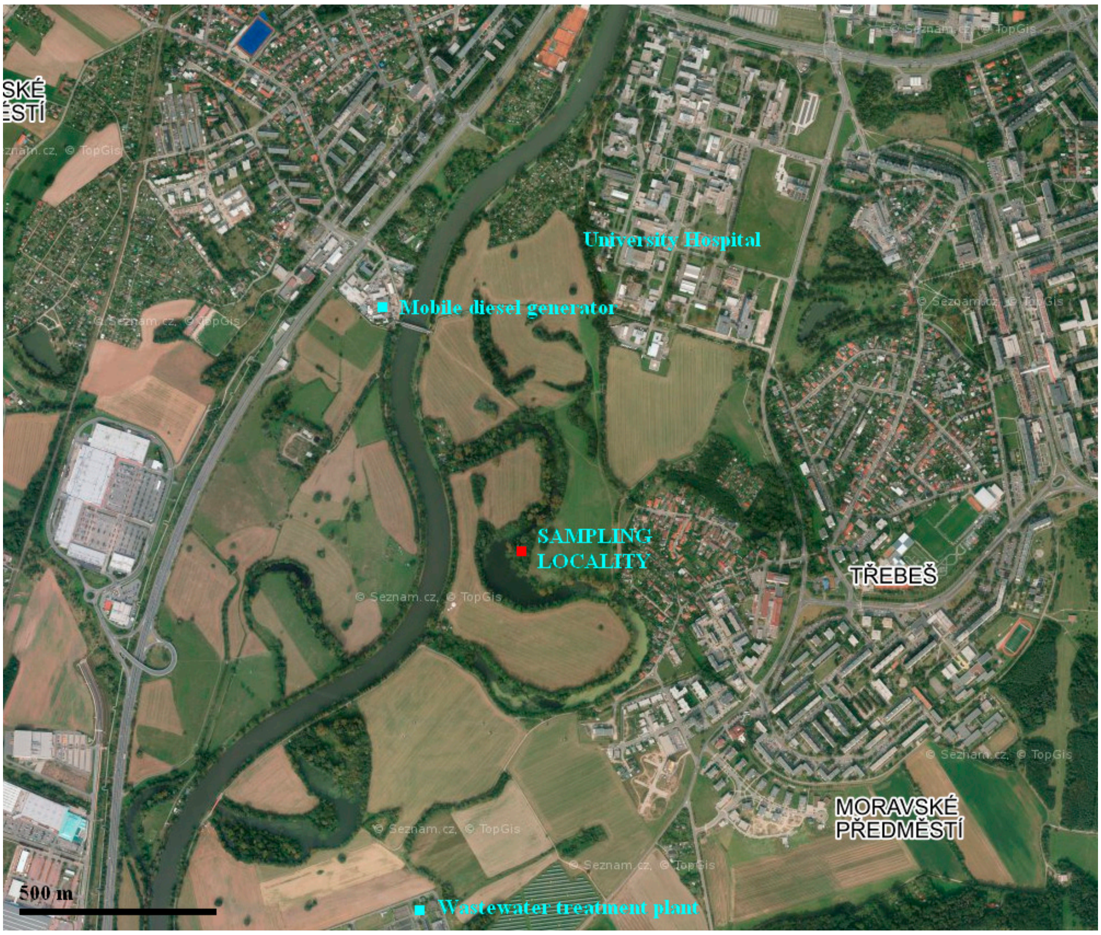

Supplement: Supplementary file 1 [file foods-10-01608-s001.zip › foods-1213579-SI.pdf]
